# The Results in 178 Cases of Prefrontal Leucotomy at the Bristol Mental Hospitals

**Published:** 1952-10

**Authors:** Edward H. Hare

**Affiliations:** Assistant Psychiatric Physician, Bristol Mental Hospital, Barrow Gurney


### THE RESULTS IN 178 CASES OF PREFRONTAL LEUCOTOMY AT THE BRISTOL MENTAL HOSPITALS

BY

EDWARD H. HARE, M.A., M.D., D.P.M.

*Assistant Psychiatric Physician, Bristol Mental Hospital, Barrow Gurney*

### 1. INTRODUCTION

The operation of prefrontal leucotomy as a means of treating mental disorder was first introduced by Moniz in 1935. In Bristol, the first leucotomy was performed in 1941 and during the following decade the operation was done on 178 psychiatric cases in the Bristol Mental Hospitals. The present paper summarizes the results obtained in these cases.

### 2. MATERIAL

The criteria for selection of cases for leucotomy have been those commonly adopted in Great Britain: mental disorder which has not responded to other methods of therapy, with negligible chances of spontaneous improvement and usually with preservation of emotional tension or of interest in social intercourse. In all but two of the 178 cases, the standard prefrontal leucotomy was performed.

Tables I and II indicate the material classified by diagnosis and age-groups. Of the thirty-two cases classed as affective, twenty-nine were in chronic depression; the remaining three had shown both manic and depressive symptoms. Of the thirteen cases grouped as Other Disorders, ten were neurotic, two post-encephalitic, and one a defective psychopath.

### 3. METHOD OF RECORDING RESULTS

Kolb (1949) in a criticism of leucotomy follow-up studies, concludes that the most satisfactory method of reporting results is by recording the patient's condition at a definite time after operation (e.g., result after one, three, or five years). This method has the approval of Penrose (1944). Further, Kolb states that assessments of " recovered ", " improved ", " social failure ", etc. are vague, subjective, and often second-hand; the only satisfactory methods of assessment are (1) detailed case reports, or (2) the bare fact whether or not the patient has been discharged from hospital.

In the present report, the state of patients is recorded one year after leucotomy (in 178 cases) and five years after (in the first 100 cases). Each patient's state is recorded as one of five grades, as shown in Table III.

### 4. RESULTS

The one-year results in 178 cases are shown in Table III. Of the patients classed as " discharged relieved ", four were discharged against medical advice

TABLE I

*Diagnoses*

| Diagnosis | Male | Female | Total | Per cent |
| --- | --- | --- | --- | --- |
| 1. Schizophrenia: .. .. | 52 | 81 | 133 | 75 |
| simple and hebephrenic | 12 | 21 | 33 | |
| catatonic .. .. | 9 | 26 | 35 | |
| paranoid .. .. .. | 22 | 31 | 53 | |
| other .. .. .. | 9 | 3 | 12 | |
| 2. Affective .. .. .. | 12 | 20 | 32 | 18 |
| 3. Other .. .. .. | 7 | 6 | 13 | 7 |
| Totals .. .. .. | 71 | 107 | 178 | 100 |

TABLE II

*Age-groups*

| Age-group | Schizophrenic | Affective | Other | Total |
| --- | --- | --- | --- | --- |
| Up to 19 | 2 | — | — | 2 |
| 20 to 29 | 27 | — | 1 | 28 |
| 30 to 39 | 61 | 2 | 7 | 70 |
| 40 to 49 | 31 | 6 | 4 | 41 |
| 50 to 59 | 10 | 17 | 1 | 28 |
| 60 to 69 | 2 | 7 | — | 9 |
| *Average age:* | 42 *years* | 56 *years* | 44 years | 45 *years* |

TABLE III

*One-year results in* 178 *cases*

| Result | Total | Percentage |
| --- | --- | --- |
| Grade 1. Discharged recovered .. .. .. | 16 | 9·0 |
| ,, 2. ,, relieved .. .. .. | 38 | 21·5 |
| ,, 3. In hospital improved .. .. .. | 63 | 35·0 |
| ,, 4. ,, unchanged .. .. .. | 55 | 31·0 |
| ,, 5. Dead .. .. .. .. .. | 6 | 3·5 |
| Totals .. .. .. .. .. .. | 178 | 100·0 |

and recorded in the notes as " not improved ". The fifty-five patients classed as "in hospital unchanged" includes two who were worse than before operation.

Table IV shows the results of 100 cases at intervals of one, three and five years after operation.

By the end of 1951, thirteen deaths had occurred among the 178 cases of leucotomy, and of these five were directly attributable to operation (operational mortality 2·8 per cent). The ages at operation of these five cases were between 30 and 50; there have been no operational deaths among the thirty-seven patients aged over 50.

Five patients (four schizophrenics and one psychopath) have had post-operative epileptic fits (3·3 per cent). Only one of these patients has had more than three fits.

TABLE IV

*One-, three- and five-year results in* 100 *cases*

| Result | Schizophrenic | | | Affective | | | Total | | |
|---|---|---|---|---|---|---|---|---|---|
| Years | 1 | 3 | 5 | 1 | 3 | 5 | 1 | 3 | 5 |
| 1. Discharged recovered .. | 4 | 8 | 8 | 8 | 9 | 9 | 12 | 17 | 17 |
| 2. ,, relieved .. | 8 | 10 | 13 | 3 | 5 | 4 | 13 | 16 | 17 |
| 3. In hospital improved .. | 32 | 25 | 21 | 5 | 2 | 1 | 37 | 29 | 24 |
| 4. ,, ,, unchanged .. | 27 | 26 | 26 | 8 | 8 | 9 | 36 | 34 | 36 |
| 5. Dead .. .. .. | 2 | 4 | 5 | — | — | 1 | 2 | 4 | 6 |
| Totals .. .. .. | | 73 | | | 24 | | | 100 | |
| Per cent discharged .. | 16 | 25 | 29 | 46 | 58 | 54 | 25 | 33 | 34 |

5. FACTORS INFLUENCING THE RESULTS

*Age.* This cannot be assessed from the present series, as the numbers are too small to allow the factor of age to be separated from that of diagnosis.

*Sex.* A higher proportion of males than of females was discharged, but again the factor of sex cannot be separated from that of diagnosis.

*Diagnosis.* The one-year results among different diagnostic categories are shown in Table V.

*Duration of illness.* Table VI shows the relation between duration of illness at the time of operation and the one-year result in schizophrenics. The proportion of patients discharged from hospital within a year after operation falls considerably when the duration of the previous illness exceeds four years. On the other hand, the proportion of patients who are unchanged by operation is not much influenced by duration of illness. Thus it may be said that while the proportion of patients improved by operation (60–70 per cent) is independent of the duration of illness, the degree of improvement within a year is in inverse ratio to this duration. A similar though less-marked relation between result and duration of illness was found in the twenty-nine cases of chronic depression.

TABLE V

*One-year results by diagnostic categories*

| Result | Schizophrenics | | | | | Affective | Other |
|---|---|---|---|---|---|---|---|
| | Hebephrenic | Catatonic | Paranoid | Other | Total | | |
| 1. Discharged recovered | 2 | — | 3 | 1 | 6 | 9 | 1 |
| 2. ,, relieved | 8 | 3 | 12 | 1 | 24 | 5 | 9 |
| 3. In hospital improved | 11 | 19 | 20 | 4 | 54 | 8 | 1 |
| 4. ,, ,, unchanged | 12 | 12 | 15 | 5 | 44 | 10 | 1 |
| 5. Dead .. .. | — | 1 | 3 | 1 | 5 | — | 1 |
| Total .. .. | 33 | 35 | 53 | 12 | 133 | 32 | 13 |
| Per cent discharged | 30 | 9 | 30 | — | 22·5 | 44 | — |

TABLE VI

*Relation between pre-operative duration of illness and one-year result in* 133 *cases of schizophrenia*

| Duration of illness before leucotomy (years) | One-year result | | | No. of cases |
|---|---|---|---|---|
| | Discharged | In hospital improved | In hospital unchanged | |
| | % | % | % | |
| Less than 2 .. | 41 | 17 | 41 | 29 |
| 2–4 .. .. .. | 33 | 33 | 30 | 33 |
| 4–9 .. .. .. | 14 | 48 | 33 | 42 |
| More than 9 .. | 7 | 59 | 28 | 29 |

Table VII shows that late improvement (sufficient for discharge from hospital) tended to occur especially in those cases with long pre-operative illness. Thus, although there was a clear-cut relation between the one-year discharge rate and duration of illness, this relation became progressively less marked as the time after operation increased. This finding may explain the conflicting reports of different authors as to the effect of duration of illness on the result of leucotomy, for these reports have been based on the aggregate result of cases assessed at varying intervals of time after operation (see, for example, Gillies *et al.* 1952).

TABLE VII

*Relation between pre-operative duration of illness and the one-, three- and five-year discharge rates in 73 cases of schizophrenia*

| Duration of illness before leucotomy (years) | No. of patients discharged after a post-operative period of | | | Total no. of cases |
|---|---|---|---|---|
| | 1 year | 3 years | 5 years | |
| Less than 2 .. | 2 | 3 | 3 | 8 |
| 2–4 .. .. .. | 5 | 5 | 5 | 18 |
| 4–9 .. .. .. | 4 | 8 | 10 | 26 |
| More than 9 .. | 1 | 2 | 3 | 21 |

*Response to electric convulsion and coma insulin therapy.* Gillies *et al.* (1952) found that patients who made a good, if transient, response to E.C.T. made a much better response to subsequent leucotomy than those who made no response to E.C.T. No such relation was found in the present series of cases (Table VIII), nor was any relation found between response to insulin coma and the effect of subsequent leucotomy.

TABLE VIII

*Relation between response to E.C.T. and to leucotomy in 67 cases*

| Response to E.C.T. | Response to leucotomy | | | |
|---|---|---|---|---|
| | Discharged | | In hospital | |
| | Recovered | Relieved | Improved | Unchanged |
| Improved—36 .. | 2 | 10 | 7 | 17 |
| Not improved—31 | 3 | 10 | 8 | 10 |

*Time of first post-operative improvement.* Table IX shows that of the improvements the great majority (85 per cent) occurred within the first month after operation, none after a year. Eighty per cent of discharges occurred in the first year, 90 per cent within the first two years. Improvement, apparently spontaneous, may occur long after leucotomy. Thus a patient with a nine-years history of chronic depression underwent leucotomy, shewed a slight improvement for six months and then relapsed to his former condition; seven years later he began to improve again, was discharged, and has remained well and working at his old job for two years.

*Relapses.* Of the sixty-two (among 178) cases discharged during the first post-operative year, eight (seven schizophrenics, one affective) relapsed, i.e. were re-admitted. This gives a one-year relapse rate of 13 per cent. Among the 100 cases studied for five post-operative years, forty were discharged, and of these five had been readmitted for a period of at least twelve months, while two others had been readmitted for periods less than twelve months. Thus the five-year relapse rate was 18 per cent. Comparison of the one-year and five-year relapse rates

TABLE IX

*Relation between time after operation and first improvement or relapse*

| Time after operation | Improvements | | Relapses | | No. of Cases |
|---|---|---|---|---|---|
| | 1st improvement in hospital | 1st discharge | 1st relapse in hospital | 1st readm. | |
| 0–1 month | 106 | 4 | — | 0 | 178 |
| 1–3 ,, | 9 | 13 | 9 | 0 | |
| 3–6 ,, | 7 | 29 | 5 | 2 | |
| 6–12 ,, | 3 | 19 | 9 | 6 | |
| 1–2 years | 0 | 8 | 2 | 2 | 150 |
| 2–3 ,, | 0 | 3 | 0 | 2 | 136 |
| 3–5 ,, | 0 | 4 | 0 | 3 | 100 |
| 5–7 ,, | 0 | 1 | 0 | 4 | 60 |

reveals no tendency to late relapse; on the contrary, the longer a patient has been discharged, the more likely he is to continue well.

### 6. DISCUSSION

The therapeutic value of leucotomy is a subject on which British reports have been silent. It seems probable indeed that leucotomy as a therapeutic procedure has already passed the stage where a scientific evaluation by a control series is feasible. Kolb (1949) concludes that " at the present time the evidence is quite inadequate to lead to the conclusion that lobotomy has therapeutic value in the treatment of schizophrenic reactions, though it may be effective in causing the remission of specific symptoms." Worthing *et al.* (1951) have made an attempt at evaluation by comparing the discharge rate of a series of leucotomized patients with those of a diagnostically comparable series studied before the introduction of modern methods of treatment; they conclude that the operation results in at least a 10 per cent increase in the number of discharges. However, their comparison is not altogether satisfactory, as the discharge rate of psychiatric patients has varied widely in different hospitals and during different decades (Prichard 1835, Solomon 1950), and is probably associated more with changes in diagnostic fashion and local and temporal variation in the enthusiasm of the medical and nursing staff than in the value of special therapeutic measures.

The results in the Bristol series correspond closely with those of most other comparable series (Board of Control 1947, Stengel 1950, Worthing *et al.* 1951, Gillies *et al.* 1952); and although the conclusion that leucotomy effects a near-recovery in 30 per cent of otherwise hopelessly chronic patients may, in the light of Kolb's criticisms, be somewhat too sanguine, yet the general impression gained from a study of the Bristol series is in conformity with that of other British reports, namely, that the operation is on the whole of value.

It must be remembered that as experience is gained the indications for leucotomy are becoming continually better defined. The disadvantages (especially among schizophrenics) of unduly delaying operation are apparent, while the suitability of leucotomy as a treatment for certain types of neurosis is becoming established. It is therefore reasonable to expect that future series will show results considerably better than those recorded here.

### 7. SUMMARY

The results in the first 178 cases subjected to leucotomy at the Bristol Mental Hospitals are summarized. It is shown that there are advantages in expressing the results in terms of the patient's condition at a definite time after operation. No relation was found between pre-operative response to electric convulsion treatment and the subsequent response to leucotomy.

The Bristol results are in close conformity with other comparable series.

### REFERENCES

Board of Control Report, (1947) *Prefrontal Leucotomy in* 1,000 *Cases.*, H.M.S.O.

Gillies, H., Hickson, B., and Mayer-Gross, W., (1952) *Brit. Med. J.* **1**, 527.

Kolb, L. C., (1949) *J. Nerv. Ment. Dis.*, **110**, 112.

Penrose, L. S., (1944) Ontario Department of Health. *Results of Special Therapies in Ontario Hospitals.*

Prichard, J. C., (1835) *A Treatise on Insanity*, p. 139. London.

Solomon, H. C., (1950) *Digest. Neurol. Psychiat.* **18**, 635.

Stengel, E., (1950) *J. Ment. Sci.* **96**, 633.

Worthing, H. J., Brill, H., and Widgerson, H. (1951) *Amer. J. Psychiat.*, **108**, 328.

